# Causes of fatalities in motor vehicle occupants: an overview

**DOI:** 10.1007/s12024-022-00503-3

**Published:** 2022-07-26

**Authors:** Siobhan O’Donovan, Corinna van den Heuvel, Matthew Baldock, Roger W. Byard

**Affiliations:** 1 School of Biomedicine, The University of Adelaide, Level 2 Helen Mayo North Building, Frome Road, Adelaide, SA 5005 Australia; 2 Forensic Science SA, Adelaide, Australia; 3 Centre for Automotive Safety Research, Adelaide, Australia; 4 The University of Adelaide, Adelaide, South Australia Australia

**Keywords:** Vehicle crash, Blunt force trauma, Sharp force and penetrating trauma: incineration, Drowning, Asphyxia

## Abstract

Injuries from motor vehicle collisions are frequently encountered in routine forensic practice. While the most common lethal events involve blunt force trauma with injuries to the head and neck, chest, abdomen, pelvis and limbs, review of the literature and case files shows that a wide variety of other fatal situations can occur that may involve sharp force and penetrating trauma, incineration, drowning, asphyxia, organic diseases and combinations of these. The following overview details potential factors that may contribute to death following vehicle crashes.

## Introduction

Motor vehicle collisions (MVCs) remain a leading cause of morbidity and mortality globally [1] and continue to occupy a considerable portion of medico-legal caseloads. Motor vehicle occupants comprise a substantial proportion of road fatalities [1]. Injuries most commonly sustained by motor vehicle occupants are usually caused by blunt force trauma although deaths may also involve penetrating and sharp force trauma, fire, drowning, asphyxia and organic diseases. The following overview outlines potential factors identified at autopsy that may contribute to death in occupants involved in MVCs.

## Classification of deaths

### Blunt force

Blunt force trauma is the leading cause of death for motor vehicle occupants with head injury being the most common lethal event [2, 3]. Head injuries may involve focal or diffuse trauma with primary impacts to the head causing skull fractures and cerebral haemorrhage, contusions and lacerations [4]. Depressed skull fractures or basilar fractures (including hinge and ring fractures) are often life-threatening given the potential for associated cerebral injury [5]. Basal-frontal skull fractures often have concomitant facial fractures which, although usually not life-threatening, may compromise airway integrity and/or cause significant haemorrhage. Axial rotational forces and angular acceleration, usually in combination with head impact, may cause shearing of axonal connections with diffuse axonal injury (DAI) [5, 6]. Mild forms of DAI may be difficult to recognise clinically and a neuropathological examination may be required for confirmation at autopsy. However, severe forms of DAI may produce visible lesions that involve the brainstem or corpus callosum [7].

Spinal cord injury is not uncommon in vehicle collisions and is often accompanied by vertebral fracture and/or dislocation. Upper cervical fractures are associated with the rapid hyperextension of the neck caused by extreme deceleration or chin contact with the instrument panel or steering wheel [8]. The atlanto-occipital joint is particularly vulnerable to fracture and dislocation during neck hyperextension which can cause rupture or tearing of the brainstem and/or spinal cord [9, 10]. Functional decapitation may also occur if the skin remains intact but the internal neck structures are disrupted below the occiput [11]. Decapitation may also be a result of vehicle-facilitated suicide by ligature strangulation whereby a ligature is tied to a fixed object, usually a tree, with the other end around the individual’s neck and then the vehicle is driven away at a high speed [11–13].

Blunt chest trauma, often from impact with the vehicle interior, results in a cluster of thoracic injuries, some of which are immediately lethal. Fatal injuries to the heart include full thickness lacerations with penetration of the atrial or ventricular walls or, in extreme cases, complete avulsion. A phenomenon known as commotio cordis may also occur in cases of significant non-penetrating chest impact with no observable cardiac injury [14]. In this situation, impact may disrupt the electrophysiological rhythm of the heart and cause immediate cardiac arrest [15].

Lethal aortic injuries such as transection or avulsion cause rapid blood loss with death typically occurring at the scene of the collision [16]. Multiple rib fractures commonly occur following blunt chest trauma and are frequently associated with injuries to the lungs and intra-thoracic vessels [17]. Three or more consecutive rib fractures at multiple locations in the same ribs can produce a flail chest which results in paradoxical movement of the flail segment during respiration [18]. While the paradoxical movement reduces pulmonary efficiency, associated lung contusions may also greatly reduce pulmonary perfusion and gas exchange [19]. Rib fractures from vehicle-related trauma must be distinguished from injuries sustained during attempted resuscitation [18, 20, 21].

Injuries to highly vascularised organs in the abdomen such as the liver, spleen and kidneys may result in significant blood loss, and in the setting of multiple trauma, abdominal injury may contribute to hypovolemic shock and exsanguination [22]. Significant torso injuries are frequently associated with pelvic fractures and destabilisation of the pelvic ring which can also damage major vessels, further contributing to blood loss [23].

Catastrophic blunt force trauma causing substantial body/organ disruption commonly results from collisions characterised by high impact speeds or impacts with objects larger than the vehicle itself, such as heavy vehicles or trains. Cases of whole body transection, typically occurring horizontally across the abdomen or pelvis, have also been reported and are often associated with severe damage to the vehicle cabin [24].

On occasion seat belts may be responsible for serious fractures, organ injuries and vessel transection, particularly in high speed collisions [25, 26].

### Sharp force and penetrating injuries

Sharp force (SF) or penetrating trauma typically occurs concurrently with blunt force injuries [27, 28]. SF injuries may be caused by broken glass from the windscreen and windows [29] and penetrating injuries are caused by loose, airborne objects from inside the vehicle or external objects that enter the vehicle cabin during the collision [30]. Fatal penetrating injuries have also been reported from the malfunction and inappropriate deployment of airbags [31, 32]. Impalement is a severe form of penetrating injury characterised by an object penetrating the body [27]. Objects causing impalement are often from parts of the external road environment such as wooden guard rails or metal fences, or tree branches [27, 28, 33–35].

### Incineration and inhalation of products of combustion

Incineration may occur as the primary cause of death or may follow lethal injury after a collision. As occupants are often either able to self-extricate from a vehicle or are removed by bystanders prior to the vehicle catching alight, incineration is not a common event [36]. A carboxyhemoglobin (COHb) saturation above 30% and/or the presence of soot in the airways at autopsy are in keeping with survival for some time after the collision [37]. Severe charring and thermal injuries may complicate pathological examination as differentiating traumatic from heat-related fractures may be difficult, especially when there is overlap [38, 39]. Incineration may also mask or destroy soft tissue and organ damage, making it difficult to ascertain the range of possible lethal blunt force injuries.

### Drowning

Drowning when vehicles enter water is uncommon, as a driver who is not incapacitated can usually escape from the cabin. Driver incapacitation may, however, occur as a result of an acute medical episode, intoxication or due to loss of consciousness or severe injuries from the impact. In floods, drivers may either be trapped within a vehicle cabin or may be drowned in fast flowing currents once the vehicle has been exited. The possibility of a vehicle-assisted suicide should also be considered when a vehicle has apparently been deliberately driven into a body of water.

### Asphyxia

There are several types of asphyxial events which may compromise tissue oxygenation that include thoracic compression [40], neck compression or an abnormal body position [41–43]. Crush asphyxia refers to mechanical compression of the thorax, common in roll-over crashes with complete or partial occupant ejection resulting in a vehicle landing on top of the occupant compressing the thorax [40]. Thoracic compression may result in fractures to the ribs or sternum, lung and vascular injuries and other injuries associated with blunt force trauma [40]. Accidental asphyxia due to hanging is almost exclusively the result of a seat belt slipping over the thorax onto the neck and may have concomitant neck injuries such as ligature markings or fracture of the hyoid bone or thyroid cartilage [43]. “Submarining” under a seat belt may occur in children or people with smaller stature [44–46]. Suicidal hanging from seat belts has been reported [47]. Positional or postural asphyxia may occur if an occupant is placed in a position which impedes respiration through neck flexion, inversion or suspension from a seat belt [41]. Positional asphyxia may occur without injury; however, classic pathological features of asphyxia including facial petechial haemorrhage, particularly of the conjunctiva of lower eyelids, may be present [40, 42, 43].

Death from asphyxia may be associated with concomitant head injuries, other blunt force trauma and alcohol/drug intoxication as these may reduce the level of consciousness and interfere with attempts at self-extrication [41, 48, 49].

### Toxicity

Death caused by carbon monoxide (CO) toxicity in vehicles is most often the result of fire or deliberate exposure, usually from car exhaust. Although rare, cases of accidental CO toxicity have been reported, often involving leaking exhaust systems [50–53]. In these cases, the odourless, tasteless and colourless nature of the CO meant that the leaks went undetected.

Byard et al. reported three cases of suspected gasoline toxicity following vehicle roll-overs with damage to fuel tanks. Volatile hydrocarbons were detected in post-mortem blood and corrosive external burns were documented associated with exposure to gasoline [54].

### Natural disease

Certain underlying diseases may precipitate or cause a crash by impacting driving ability, perception and consciousness. Medical episodes include (but are not limited to) stroke, myocardial infarction/ischemic heart disease, pulmonary embolism, abdominal aortic aneurysm (AAA) rupture, seizures, hypoglycaemia, dementia and mental illness [55–59]. Several of these, such as myocardial infarction, stroke or rupture of an aortic aneurysm, may have warning signs enabling a driver to pull over or to reduce vehicle speed before losing consciousness [58]. However, seizure-related conditions such as epilepsy or syncopal episode may rapidly incapacitate a driver [60].

Organic underlying disease may also contribute to death following trauma in the elderly as the body’s physiological response to trauma is reduced with age, often with the presence of other age-associated diseases [61].

### Sequelae of injury

Improvements in pre-hospital care and emergency responses have greatly enhanced crash survivability but there are several injury sequelae that can contribute to mortality following a collision.

- Secondary injury mechanisms from TBI such as cerebral ischemia, hypoxia, oedema and raised intracranial pressure may be fatal despite surgical or pharmacological intervention [62, 63].
- Cerebral swelling and neurogenic pulmonary oedema from acute head trauma may occur very rapidly following head trauma [64].
- Fractures, particularly of long bones, may produce fat emboli.
- Vertebral or cerebral artery dissection may occur.
- Multi-organ failure, sepsis and hospital-acquired infections such as bronchopneumonia may develop [3].

## Conclusion

This overview has demonstrated that a wide variety of immediate and delayed lethal mechanisms may complicate vehicle crashes. At autopsy, the possibility of the additive effects of several disparate lethal mechanisms should therefore be considered.

## Key points

1. Motor vehicle crashes comprise a significant portion of medico-legal caseloads.
2. Blunt force trauma is the most common lethal event frequently with a combination of injuries to the head, chest, abdomen and limbs.
3. A variety of other fatal situations may occur that may involve sharp force and penetrating trauma, incineration, drowning, asphyxia, organic diseases or combinations of these.
4. Mechanism may be immediately lethal or death may occur from post-trauma sequelae.
